# Influenza-associated mortality in Thailand, 2006–2011

**DOI:** 10.1111/irv.12344

**Published:** 2015-10-13

**Authors:** Suchunya Aungkulanon, Po-Yung Cheng, Khanitta Kusreesakul, Kanitta Bundhamcharoen, Malinee Chittaganpitch, McCarron Margaret, Sonja Olsen

**Affiliations:** a International Health Policy Program, Ministry of Public HealthNonthaburi, Thailand; b Influenza Division, Centers for Disease Control and PreventionAtlanta, GA, USA; c Naitonal Institute of HealthNonthaburi, Thailand; d Thailand Ministry of Public Health - United States Centers for Disease Control and Prevention CollaborationNonthaburi, Thailand

**Keywords:** Influenza, mortality, Thailand, tropical

## Abstract

**Background:**

Influenza-associated mortality in subtropical or tropical regions, particularly in developing countries, remains poorly quantified and often underestimated. We analyzed data in Thailand, a middle-income tropical country with good vital statistics and influenza surveillance data.

**Methods:**

We obtained weekly mortality data for all-cause and three underlying causes of death (circulatory and respiratory diseases, and pneumonia and influenza), and weekly influenza virus data, from 2006 to 2011. A negative binomial regression model was used to estimate deaths attributable to influenza in two age groups (<65 and ≥65 years) by incorporating influenza viral data as covariates in the model.

**Results:**

From 2006 to 2011, the average annual influenza-associated mortality per 100 000 persons was 4·0 (95% CI: −18 to 26). Eighty-three percent of influenza-associated deaths occurred among persons aged > 65 years. The average annual rate of influenza-associated deaths was 0·7 (95% CI: −8·2 to 10) per 100 000 population for person aged <65 years and 42 (95% CI: −137 to 216) for person aged ≥ 65 years.

**Discussion:**

In Thailand, estimated excess mortality associated with influenza was considerable even during non-pandemic years. These data provide support for Thailand's seasonal influenza vaccination campaign. Continued monitoring of mortality data is important to assess impact.

## Introduction

The global burden of influenza is unknown but thought to be considerable. In 2008, the global estimate of influenza-associated severe acute lower respiratory illness in children <5 years was 1 million cases,^1^ and the World Health Organization (WHO) suggests that 3–5 million severe cases occur in persons of all ages each.^2^ In temperate regions, influenza usually causes annual outbreaks during the winter season (November–March in the Northern Hemisphere and June–September in the Southern Hemisphere).^3^,^4^ In contrast, influenza-associated mortality in subtropical or tropical regions, particularly in developing countries, remains poorly quantified and often underestimated. The pattern of influenza epidemics in tropical regions is less distinct and more diffuse. Seasonal influenza epidemics can sometimes occur twice or more throughout a year.^5^

The exact burden of influenza morality is difficult to estimate, and the challenges in counting influenza-associated deaths include the following: testing of hospital patients for influenza (particularly in elderly persons) is uncommon, influenza is rarely specifically recorded on death certificates, and many deaths that may be causally related to influenza occur after virus can be detected. Therefore, estimation of influenza-associated deaths or hospitalizations often relies on statistical modeling rather than on direct measurement. Various methodological approaches have been used to estimate the excess deaths associated with the circulation of influenza virus in temperate region.^6^–^10^ However, the best approach for modeling influenza in tropical regions is unclear. Further, although there are results for wealthy, tropical countries (e.g., Singapore),^11^ the findings may be different from Thailand, where the severity of infections may be compounded by a higher prevalence of underlying illness or other factors.. In this study, we used a regression model to examine the impact of influenza, by virus type and subtype, on deaths in Thailand, while adjusting for potential confounding effects by other co-circulating influenza virus subtypes.

Estimating the burden of influenza mortality is important to help guide vaccination programs, evaluate the use of diagnostic tests and antiviral drugs, and plan for seasonal epidemics and future pandemics. In this analysis, we applied a negative binomial model to the weekly counts of deaths and viral data to explore the seasonal effect of influenza on mortality and provided estimates of excess mortality associated in Thailand by death category, age group, and influenza subtypes for the years 2006 through 2011.

## Methods

### Mortality data

Weekly electronic mortality data for years 2006 through 2011 were obtained from the Bureau of Policy and Strategy, Ministry of Public Health (MoPH), Thailand. Deaths were categorized into three groups based on codes from the International Classification of Diseases, Tenth Revision [ICD-10]: circulatory diseases (*ICD-10* codes I00-99), respiratory diseases (ICD-10 codes J00-99), and pneumonia and influenza (P&I) (ICD-10 codes J10-18). Other variables included age at death and location of death (hospital or community). We defined non-pandemic years as every year except 2009.

### Viral surveillance data

In 2004, the National Institute of Health, MoPH launched the national influenza sentinel surveillance system to monitor virus circulation in patients with influenza-like illness presenting at outpatient clinics in 11 sites throughout Thailand.^12^ Between 2004 and 2009, each site was first instructed to enroll a convenience sample of up to five patients per week with ILI for a total of 20 patients per month. In September 2009, the sample size was increased to 10 patients per week (five from children aged <15 years and five from persons aged ≥ 15 years). These data, collected systematically throughout the year, represent an unbiased sample of the timing of influenza activity and are appropriate for analyzing seasonal trends. All specimens were tested by reserve-transcription polymerase chain reaction for influenza viruses. We obtained the weekly numbers of influenza viruses (by type and subtype) and the total number of specimens tested, from the National Institute of Health, and calculated weekly positivity using weekly number of specimens as the denominator.

### Reapportioning mortality data

Of all-cause deaths among person aged <65 and ≥65 years, ill-defined deaths (i.e., no cause listed) accounted for 25% (58% of these occurred in the community) and 54% (87% occurred in the community), respectively. To account for the high proportion of ill-defined cause of death, we reapportioned weekly ill-defined deaths by age (<65 and ≥65 years), and location (hospital and community) to the various categories (P&I, respiratory, circulatory). We first excluded the ill-defined deaths and calculated the proportion of deaths due to P&I, respiratory and circulatory among deaths recorded in-hospital by age (<65 and ≥65 years) under the assumption that the coding for an in-hospital death was more accurate than for a community death. This assumption is based on the knowledge that deaths occurring outside the hospital are recorded by non-medical, civil registrars and based on lay reports from relatives.^13^ We then reapportioned all ill-defined deaths (including both those that occurred in-hospital and those in the community) to the various categories (P&I, respiratory, circulatory) so that the final proportion in each category were equivalent to those found in the in-hospital deaths.

We created two separated databases, one without reapportioning the deaths and the other with reapportioning.

### Climate data

Meteorological parameters including weekly mean temperature and absolute humidity were obtained from the Thai Meteorological Department.

### Negative binomial regression model

A negative binomial regression model was used to estimate deaths attributable to influenza in two age groups (<65 and ≥65 years) by incorporating influenza viral data as covariates in the model. To assume an additive relationship between the exposure to influenza and resulting mortality, we used an identity link function in the models. The models included independent variables that comprised weekly percentage of specimens testing of confirmed infection with seasonal influenza A, seasonal influenza B, and influenza A (H1N1) pdm09 during a given week. We tested various lags (no lag, 1 week, and 2 weeks) to account for delays between infection and death. To account for the baseline, the models include either harmonic terms for annual [sine (θ) and cosine (θ), where θ = 2*π*week/52·25] and semiannual [sine (2θ) and cosine (2θ)] periods or climate factors [weekly mean temperature and absolute humidity, using linear terms or nonlinear terms (natural cubic spline function)]. Selection of the most statistically meaningful proxy for influenza activity was based on fitting 13 models to ≥65 years respiratory deaths and comparing Akaike information criterion (AIC) values. We obtained the smallest AIC values for a model with a 2-week lag and smoothing spline of temperature and humidity and report those results. A detailed description of the model fitting procedure is provided in Table S1.

In this model, 

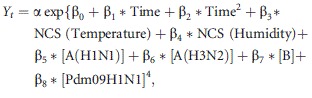
where *Y*_*t*_ is the number of deaths in week *t* and α is the offset term and is equal to the log of the population size. β_0_ represented the intercept, β_1_ and β_2_ represented coefficients associated with secular trends, β_3_ represented coefficients associated with a natural cubic spline (NCS) of weekly mean temperature, β_4_ represented coefficients associated with a NCS of absolute humidity, β_5_–β_9_ represented coefficients associated with the percentages of specimens testing positive for each influenza virus type and subtype during a given week. All viral terms had a 2-week lag. The models were run separately by death category (P&I, respiratory, and circulatory), age group (<65 and ≥65 years), and location of death (hospital or community, only for the data without reapportioned deaths). The analysis was conducted both ways, with and without reapportioning these deaths with the latter included as supplementary tables and figures.

To estimate excess mortality associated with influenza, we used the following procedure:

1. Calculate the expected mortality with the full model (E0).
2. Calculate expected mortality with the same fitted model, but the influenza terms were set as zero (E1).

E0–E1 was the estimate of mortality due to influenza.

Confidence intervals were calculated based on the standard errors of model coefficients. We note that these confidence intervals provide only a minimum estimate of uncertainty.

The statistical analyses were carried out using sas version 9.3 (SAS Institute, Cary, NC, USA).

### Sensitivity analysis

As a sensitivity analysis, we modeled all-cause deaths. We compared the excess deaths obtained using the all-cause deaths as outcome in the final models with estimates using respiratory and circulatory deaths as outcomes in the same models.

## Results

### Mortality data

From January 2006 to December 2011, an annual mean of 399 853 deaths (range, 389 696 in 2006 to 413 209 in 2011) occurred in Thailand (42% occurred in hospital). An average of 13 554 (3·4%) underlying P&I deaths, 27 268 (6·8%) underlying respiratory deaths, and 37 112 (9·3%) underlying circulatory deaths occurred each year. Table1 shows the annual mean number and proportion of deaths by cause of death. Forty-one percent of total deaths were coded as cause ill-defined and redistributed to the various categories (P&I, respiratory, circulatory) by location and age group.

**Table 1:** Average annual number and proportion of deaths by cause of death, location of death, and age group in Thailand, 2006–2011

Cause of death	Total *n* (%)	In-hospital deaths (42%)			Community deaths (58%)		
		<65 years (55%)	≥65 years (45%)	All ages	<65 years (40%)	≥65 years (60%)	All ages
Annual average number of deaths	399 853	92 178	75 962	168 139	92 519	139 195	231 714
Proportion of deaths							
Respiratory disease	27 268 (6·8)	7481 (8·1)	12 361 (16)	19 842 (12)	3575 (3·9)	3851 (2·8)	7426 (3·2)
Pneumonia and influenza	13 554 (3·4)	4583 (5·0)	7279 (9·6)	11 861 (7·1)	935 (1·0)	757 (0·5)	1693 (0·7)
Circulatory disease	37 113 (9·3)	11 118 (12)	14 482 (19)	25 600 (15)	5634 (6·1)	5879 (4·2)	11 513 (5·0)
Ill-defined	163 474 (41)	19 513 (21)	14 615 (19)	34 128 (20)	27 194 (29)	102 152 (73)	129 345 (56)

### Viral surveillance data

During the 6-year period, 21 560 specimens were tested for influenza viruses. There were 2961 (14%) positive results for influenza viruses. The annual mean number of tests positive was 14% (range 10–18%) for influenza A viruses, and 6·5% (range 4·1–10%) for influenza B viruses (Table S2).

### Estimates of influenza-associated deaths

For all ages combined, there was year-to-year variability in the annual number of influenza-associated deaths; the mean for all years was 2511 (4·0 per 100 000) (Table2). The average influenza-associated mortality rate for non-pandemic years was 4·3 per 100 000, and for the pandemic year, 2009, it was 2·4 per 100 000. The rates in pandemic year for both age groups were lower compared with the other years (<65 years: 0·6 versus 0·7 per 100 000 and ≥65 years: 24 versus 26 per 100 000). The annual death rate attributable to influenza was the highest in the year 2008 (5·6). Eighty-three percent (2094/2511) of the average annual estimated influenza-associated deaths occurred among person age ≥65 years. The average annual rate of influenza-associated deaths was 0·7 per 100 000 population for person aged <65 years and 42 for person aged ≥65 years. Among adults aged <65 years and ≥65 years, influenza A (H3N2) and B were associated with the most deaths. The average annual number of influenza-associated deaths was 1401 (2·2 per 100 000) for underlying P&I causes, 2751 (4·3 per 100 000) for underlying respiratory causes, and 529 (0·8 per 100 000) for underlying circulatory causes (Table3). Deaths among persons aged ≥65 years accounted for 74% (1031/1401), 76% (2094/2751), and 100% (529/529) of the overall estimated average annual influenza-associated deaths with underlying P&I, respiratory, and circulatory causes, respectively.

**Table 2:** Estimated annual influenza-associated deaths in Thailand, 2006–2011 (with the apportioned ill-defined deaths)

Year	A(H1N1)			A(H3N2)			Pdm09 H1N1			B			Total			Rate per 100 000		
	Death	95%CI		Death	95%CI		Death	95%CI		Death	95%CI		Death	95%CI		Rate	95%CI	
All age																		
2006	625	−3232	4151	228	−3467	3922	0	0	0	647	−3060	4328	1500	−10 351	12 993	2·4	−17	21
2007	215	−3400	3710	1866	−1690	5426	0	0	0	928	−2655	4470	2952	−8308	14 169	4·7	−13	23
2008	642	−3206	4143	1563	−2115	5240	0	0	0	1499	−2206	5150	3534	−8101	15 107	5·6	−13	24
2009	261	−3759	4137	633	−3299	4576	841	−3601	4278	356	−3593	4289	1524	−14 252	17 280	2·4	−22	27
2010	3	−4266	4271	776	−3491	5045	955	−3884	4647	1078	−3219	5312	2239	−14 860	19 275	3·5	−23	30
2011	0	−4246	4246	2273	−1965	6520	133	−4189	4299	985	−3281	5216	3315	−13 681	20 281	5·2	−21	32
Average	291	−3685	4110	1223	−2671	5122	643**	−3891	4408	916	−3002	4794	2511	−11 592	16 518	4·0	−18	26
Average (non-pandemic years)*	297	−3670	4104	1341	−2546	5231	544	−4037	4473	1027	−2884	4895	2708	−11 060	16 365	4·3	−17	26
Aged ≤ 65 years																		
2006	192	−1251	1471	25	−1337	1389	0	0	0	118	−1250	1474	335	−4430	4926	0·6	−7·7	8·5
2007	66	−1262	1332	214	−1088	1512	0	0	0	172	−1143	1460	395	−4056	4867	0·7	−7·0	8·4
2008	200	−1222	1445	180	−1158	1514	0	0	0	275	−1076	1599	485	−4030	5132	0·8	−6·9	8·8
2009	81	−1395	1487	72	−1361	1509	678	−1009	1860	66	−1377	1500	330	−5142	6356	0·6	−8·8	11
2010	1	−1547	1548	88	−1458	1639	769	−1065	2030	198	−1362	1732	483	−5432	6949	0·8	−9·3	12
2011	0	−1515	1515	259	−1250	1776	109	−1443	1581	179	−1341	1685	471	−5549	6557	0·8	−9·4	11
Average	90	−1365	1466	140	−1275	1557	519**	−1172	1824	168	−1258	1575	417	−4773	5798	0·7	−8·2	10
Average (non-pandemic years)*	92	−1359	1462	153	−1258	1566	293	−836	1204	188	−1234	1590	434	−4699	5686	0·7	−8·0	10
Aged 65+ years																		
2006	433	−1981	2680	203	−2130	2533	0	0	0	529	−1810	2854	1165	−5921	8067	25	−125	170
2007	149	−2138	2378	1652	−602	3914	0	0	0	756	−1512	3010	2557	−4252	9302	53	−89	194
2008	442	−1984	2698	1383	−957	3726	0	0	0	1224	−1130	3551	3049	−4071	9975	63	−84	206
2009	180	−2364	2650	561	−1938	3067	163	−2592	2418	290	−2216	2789	1194	−9110	10 924	24	−184	221
2010	2	−2719	2723	688	−2033	3406	186	−2819	2617	880	−1857	3580	1756	−9428	12 326	35	−185	242
2011	0	−2731	2731	2014	−715	4744	24	−2746	2718	806	−1940	3531	2844	−8132	13 724	54	−155	261
Average	201	−2320	2643	1084	−1396	3565	124**	−2719	2584	748	−1744	3219	2094	−6819	10 720	42	−137	216
Average (non-pandemic years)*	205	−2311	2642	1188	−1287	3665	105	−2783	2668	839	−1650	3305	2274	−6361	10 679	46	−128	215

* Average for non-pandemic years (excluded 2009).

** Three-year average.

**Table 3:** Annual estimated number and rate of influenza-associated deaths for underlying P&I, respiratory, and circulatory deaths in Thailand, 2006–2011 (with the apportioned ill-defined deaths)

Cause of death	Influenza					
	Number of deaths	95% CI		Rate per 1 000 000	95% CI	
Respiratory disease						
<65 years	657	−1788	3101	1·1	−0·3	5·3
≥65 years	2094	−4611	8801	42	−93	178
All ages	2751	−6400	11 902	4·3	−10·1	19
Pneumonia and influenza						
<65 years	370	−1256	2007	0·6	−2·2	3·4
≥65 years	1031	−3201	5259	21	−64·7	106
All ages	1401	−4457	7266	2·2	−7·0	11
Circulatory disease						
<65 years	0	−3363	3075	0·0	−5·8	5·3
≥65 years	529	−5934	6797	11	−120	138
All ages	529	−9297	9871	0·8	−14·7	16

**Figure 1:**
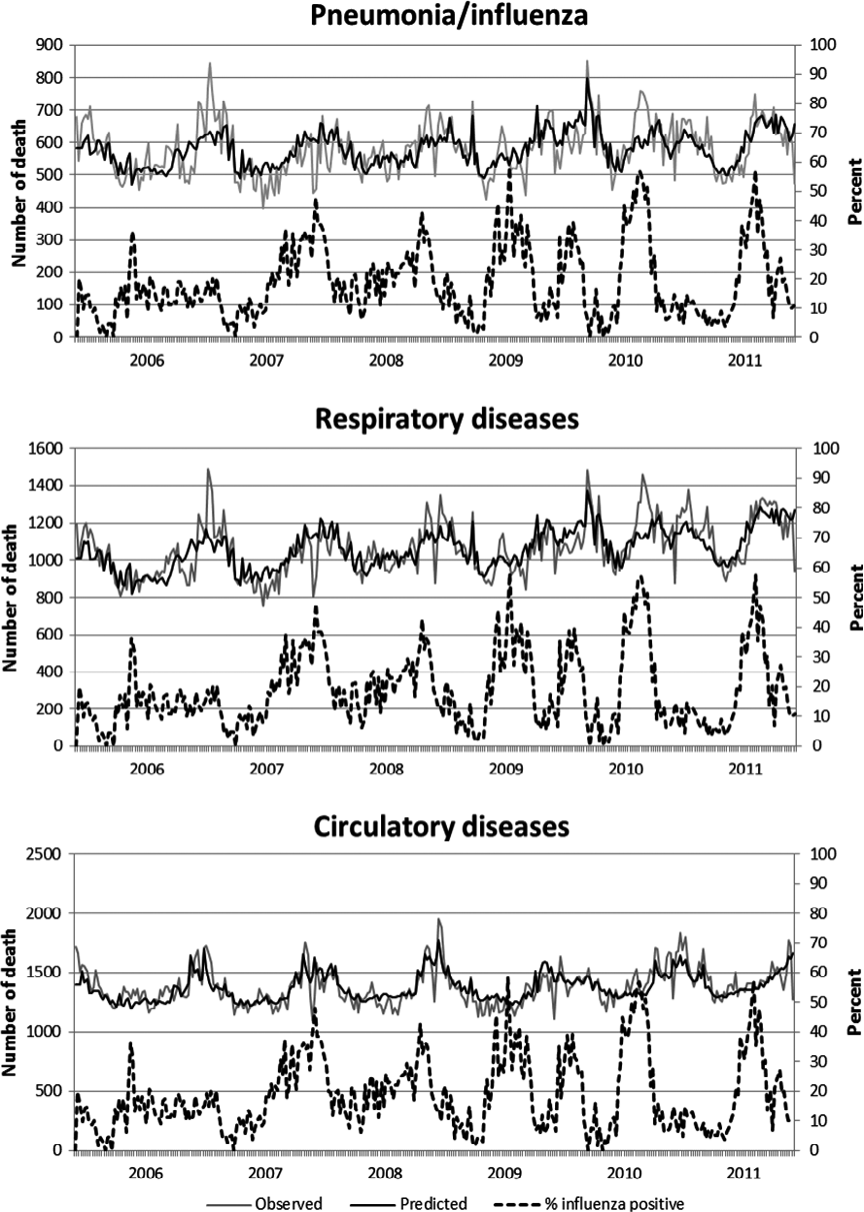
Weekly observed and predicted influenza-associated deaths from underlying P&I and respiratory and circulatory diseases (with the reapportioned ill-defined deaths).

The average annual rate of influenza-associated deaths for adults aged ≥65 years was 21 per 100 000 persons for P&I deaths, 42 per 100 000 for respiratory deaths, and 11 per 100 000 for circulatory deaths (Table3). Among adults aged <65 years, the average annual rate of influenza-associated deaths was 0·6 deaths per 100 000 persons for P&I causes and 1·1 per 100 000 for respiratory causes. The weekly number of deaths from 2006 to 2011, and excess deaths where mortality exceeded the baseline, is shown in Figure1; there are no discrete peaks indicating influenza epidemics. In the sensitivity analysis of all-cause deaths, the average annual rate of influenza-associated deaths was 2·0 (95%CI: −24 to 27) per 100 000 population for person aged <65 years and 99 (95%CI: −322 to 516) for person aged ≥65 years.

### Estimates of influenza-associated deaths without reapportion ill-defined death

Estimated mean annual influenza-associated deaths without reapportioning ill-defined death are shown in Table S3. The average annual number of influenza-associated deaths from underlying P&I, respiratory, and circulatory diseases was 632 (1·0 per 100 000), 1308 (2·1 per 100 000), and 295 (0·5 per 100 000, Table S4), respectively. The estimated number of influenza deaths from the model with reapportioned ill-defined deaths and from the non-reapportion model for persons aged <65 was similar, while for those age ≥ 65 years, the estimates from reapportioned models were approximately 3 times higher than from the non-reapportion model. Compared with Figure1, similar patterns were observed with no obvious peak for the excess circulatory and respiratory deaths and overall lower magnitude (Figure S1).

## Discussion

Influenza was associated with excess death in Thailand, and the estimated excess mortality was considerable (average 4·0 per 100 000 persons), with 83% of the influenza-associated deaths among persons ≥65 years. The estimated annual excess morality in persons ≥65 years was 42 per 100 000 persons. These finding are similar, but slightly lower than those from a recent study in Thailand that used a Bayesian model to estimate excess mortality due to seasonal influenza; this study found an estimated 6·1 annual excess deaths per 100 000 population and 68 per 100 000 among person ≥60 years.^14^ Reasons for the differences could be due to the use of different outcomes for the models; Copper and colleagues use all-cause mortality, whereas we focused on respiratory and circulatory deaths. In addition, it could also be the results of the slightly different years of data analyzed, the use of three instead of two age groups in the model, or the use of the Bayesian model.

Our estimates of annual influenza-associated underlying P&I, underlying respiratory and underlying circulatory deaths in Thailand for the all-ages group were two times lower than the estimates of excess influenza deaths observed in tropical Singapore,^11^ subtropical Hong Kong,^15^ and temperate United States.^16^ When looking by age, we observe that the decreased rates in elderly persons account for these country-specific differences. Some of these differences may be explained by the different age distribution in these countries; Hong Kong has a higher percentage of the population ≥65 years (14%) compared with Thailand (9·8%) or Singapore (8·1%).^17^ In Thailand, the proportion of influenza-associated deaths was higher among the elderly (persons ≥65 years), which is consistent with data from Hong Kong and the United States where >80% of influenza-associated deaths occurred among the elderly.^15^,^16^ However, our estimates for influenza-associated deaths in persons age ≥65 years were consistently lower than those in Singapore (46·9 per 100 000 for P&I, and 155·4 per 100 000 for circulatory and respiratory) and Hong Kong (39·3 per 100 000 for P&I and 102 per 100 000 for circulatory and respiratory).^11^,^15^ We suspect that our rates in elderly may be an underestimation resulting from inaccurate coding of death; the high proportion of ill-defined causes of death in our data set support this theory.^13^ Other factors that might have contributed to the differences in mortality impact between countries include geographical differences, age distribution, previous influenza exposure history of the population, influenza vaccination coverage, use of antiviral drugs, access to health care, and use of public health mitigation strategies. To address the issue of the high proportion of ill-defined causes of deaths in our data set, we performed a sensitivity analysis. We modeled all-cause deaths as outcome and our estimate (99 per 100 000, 95%CI: −322 to 516) for influenza-associated deaths in persons age ≥65 years was more comparable with the estimate in Singapore (167·8 (95% CI: 107·0–229·5) for all-cause) and the estimate in Hong Kong (136·1 (95% CI: 83·7–188·4) for all-cause).

Although there were peaks in influenza-associated mortality in Thailand during 2006 through 2011, they were not consistent in their timing. This is in contrast to trends observed in temperate climates and likely reflects the year-round circulation of influenza viruses which may attenuate any seasonal mortality pattern. The majority of deaths from seasonal influenza occurred among people aged 65 year or older, but in the pandemic, the proportion of deaths among younger persons increased. This may suggest that there was some immunologic protection in persons who were exposed to A(H1N1) viruses before the 1957 pandemic, as has been demonstrated in other countries.^18^,^19^

Our estimates of influenza-associated deaths have several methodological limitations. First, the quality of mortality statistics in Thailand was considered poor because a large proportion had a poorly defined cause of death.^13^,^20^ Further, the validity of the cause of death statistics was questionable as many out-of hospital deaths were coded by persons not medically qualified to determine the cause of death.^21^ Thus, misclassification of cause of death may have resulted in an underestimate of our specific mortality outcomes of interest, particularly in our excess mortality results that did not reapportion the ill-defined cause of death. Second, the relative distribution of influenza A and B viruses is known to vary by age, but the viral surveillance data were not robust enough to stratify by age and provide meaningful estimates by age group. Third, with only one cause of death listed, we were unable to assess contributing causes of death.^21^

These data are important to help guide the introduction of prevention strategies, such as seasonal influenza vaccination. Despite the widespread adoption of seasonal influenza vaccine recommendations in middle-income countries that have sufficient economic and public health resources to support vaccination programs, in Thailand seasonal influenza vaccine has been administered to <1% of the population annually and does not meet the need of the identified target groups.^22^ As vaccine use continues to increase, mortality should be monitored to assess the impact of the vaccination campaign.

## Disclaimer

The findings and conclusions in this report are those of the authors and do not necessarily represent the views of the Centers for Disease Control and Prevention or the Thailand Ministry of Public Health.

## Supporting Information

**Figure S1.** Weekly observed and predicted influenza-associated deaths from underlying P&I, respiratory and circulatory diseases (without the reapportioned ill-defined deaths).

**Table S1.** Model fitting procedure.

**Table S2.** Number of specimen tested and percent positive for influenza viruses by year, 2006–2011.

**Table S3.** Estimated average number of annual influenza-associated deaths with underlying P&I, respiratory, and circulatory causes by age group and location (without reapportion of ill-defined deaths).

**Table S4.** Estimated annual influenza-associated death rates in Thailand (without the reapportioned ill-defined deaths).
