# Supplementary figures and images for: Influenza-associated mortality in Thailand, 2006–2011
Source: Influenza Other Respir Viruses. 2015 Oct 13;9(6):298–304. doi: 10.1111/irv.12344 (PMC4605410; doi:10.1111/irv.12344)

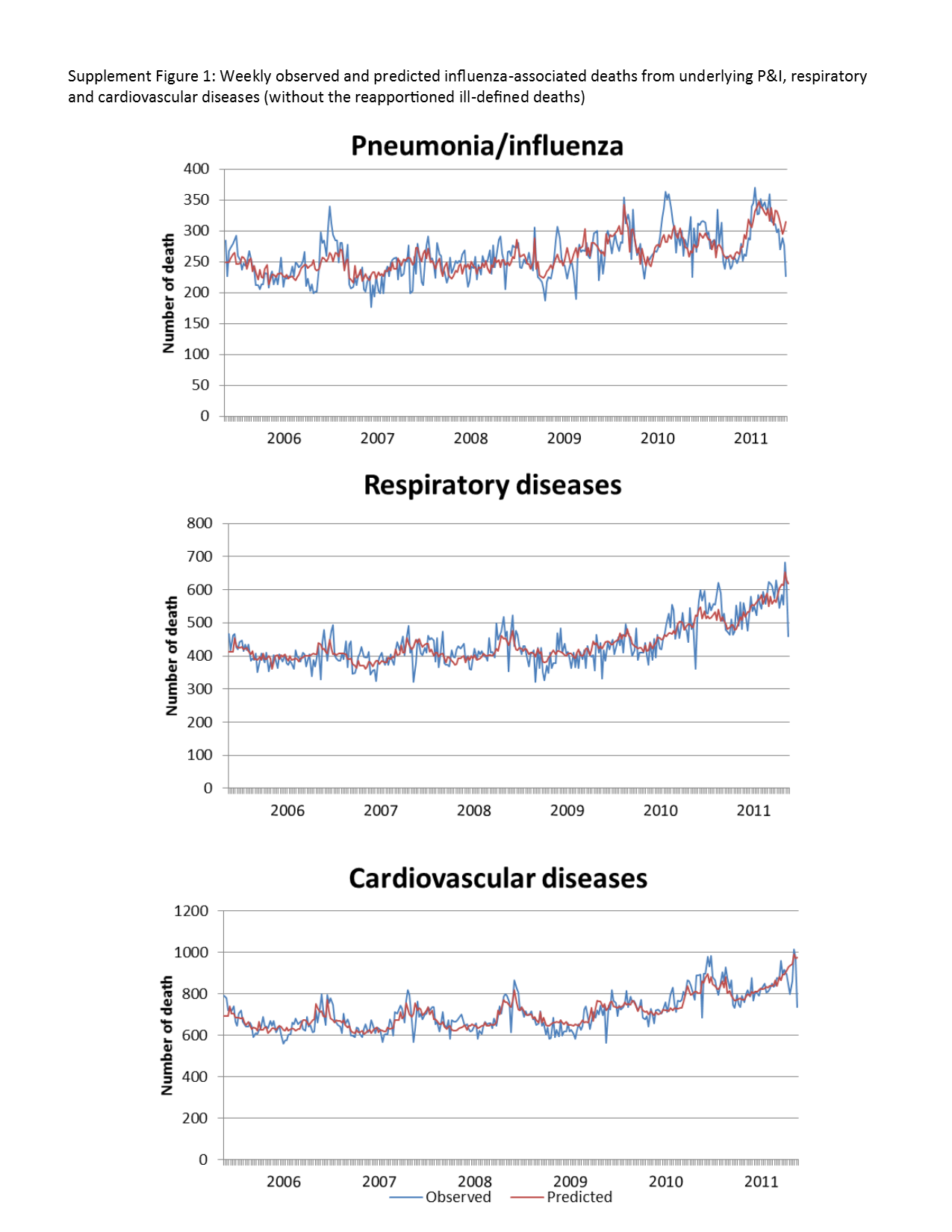

Supplement: Supplementary file 1 — Figure S1. Weekly observed and predicted influenza-associated deaths from underlying P&I, respiratory and circulatory diseases (without the reapportioned ill-defined deaths). [file irv0009-0298-sd1.tif]
