# Supplementary material for: Influenza-associated mortality in Thailand, 2006–2011
Source: Influenza Other Respir Viruses. 2015 Oct 13;9(6):298–304. doi: 10.1111/irv.12344 (PMC4605410; doi:10.1111/irv.12344)
Supplement: Supplementary file 2 — Table S1. Model fitting procedure. [file irv0009-0298-sd2.docx]

Supplement Table 1: Model fitting procedure

| Model | Time | Time^2^ | Time^3^ | SIN (2*Time*pi/52) | COS (2*Time*pi/52) | SIN (2*Time*pi/26) | COS (2*Time*pi/26) | A(H1N1) | A(H3N2) | Flu B | Pdm09 H1N1 | Temperature | Humidity | NCS(Temperature) | NCS(Humidity) | AIC |
| --- | --- | --- | --- | --- | --- | --- | --- | --- | --- | --- | --- | --- | --- | --- | --- | --- |
| 1 | x | x |  | x | x |  |  | x | x | x | x |  |  |  |  | 3668 |
| 2 | x | x | x | x | x |  |  | x | x | x | x |  |  |  |  | 3664 |
| 3 | x | x |  | x | x |  |  | x (lag 1 wk) | x (lag 1 wk) | x (lag 1 wk) | x (lag 1 wk) |  |  |  |  | 3642 |
| 4 | x | x |  | x | x |  |  | x (lag 2 wk) | x (lag 2 wk) | x (lag 2 wk) | x (lag 2 wk) |  |  |  |  | 3627 |
| 5 | x | x |  | x | x | x | x | x | x | x | x |  |  |  |  | 3652 |
| 6 | x | x |  | x | x | x | x | x (lag 1 wk) | x (lag 1 wk) | x (lag 1 wk) | x (lag 1 wk) |  |  |  |  | 3627 |
| 7 | x | x |  | x | x | x | x | x (lag 2 wk) | x (lag 2 wk) | x (lag 2 wk) | x (lag 2 wk) |  |  |  |  | 3614 |
| 8 | x | x |  |  |  |  |  | x | x | x | x | x | x |  |  | 3686 |
| 9 | x | x |  |  |  |  |  | x (lag 1 wk) | x (lag 1 wk) | x (lag 1 wk) | x (lag 1 wk) | x | x |  |  | 3651 |
| 10 | x | x |  |  |  |  |  | x (lag 2 wk) | x (lag 2 wk) | x (lag 2 wk) | x (lag 2 wk) | x | x |  |  | 3631 |
| 11 | x | x |  |  |  |  |  | x | x | x | x |  |  | x | x | 3662 |
| 12 | x | x |  |  |  |  |  | x (lag 1 wk) | x (lag 1 wk) | x (lag 1 wk) | x (lag 1 wk) |  |  | x | x | 3628 |
| 13 | x | x |  |  |  |  |  | x (lag 2 wk) | x (lag 2 wk) | x (lag 2 wk) | x (lag 2 wk) |  |  | x | x | 3612 |

The base model had time terms, viral terms (A(H1N1), A(H3N2), Flu B, and Pdm09 H1N1), and one set of either harmonic terms (SIN and COS) or climate factor terms (weekly mean temperature and absolute humidity). For viral terms, we tested the lag between infection and death (0, 1, 2 weeks). For harmonic terms, we tested one pair or two pairs of harmonic terms which reflected either one or two influenza peaks in a season. For climate factor terms, we tested the treatment of temperature and humidity as linear terms or non-linear terms (using natural cubic spline function).
