# Supplementary material for: Influenza-associated mortality in Thailand, 2006–2011
Source: Influenza Other Respir Viruses. 2015 Oct 13;9(6):298–304. doi: 10.1111/irv.12344 (PMC4605410; doi:10.1111/irv.12344)
Supplement: Supplementary file 3 — Table S2. Number of specimen tested and percent positive for influenza viruses by year, 2006–2011. [file irv0009-0298-sd3.docx]

Supplement Table 2: Number of specimen tested and percent positive for influenza viruses by year, 2006-2011

| Year | Number of specimens tested | Influenza | | | | | | | | | | | |
| --- | --- | --- | --- | --- | --- | --- | --- | --- | --- | --- | --- | --- | --- |
|  |  | A(H1N1) | | A(H3N2) | | A(H1N1)pdm09 | | A(not subtyped) | | B | | Total influenza A and B | |
|  |  | N | % | N | % | N | % | N | % | N | % | N | % |
| 2006 | 3,465 | 316 | 9.1% | 47 | 1.4% | 0 | 0.0% | 0 | 0.0% | 141 | 4.1% | 363 | 10% |
| 2007 | 4,323 | 132 | 3.1% | 345 | 8.0% | 0 | 0.0% | 0 | 0.0% | 315 | 7.3% | 477 | 11% |
| 2008 | 3,765 | 271 | 7.2% | 250 | 6.6% | 0 | 0.0% | 0 | 0.0% | 391 | 10% | 521 | 14% |
| 2009 | 3,048 | 87 | 2.9% | 78 | 2.6% | 388 | 13% | 1 | 0.0% | 80 | 2.6% | 554 | 18% |
| 2010 | 3,493 | 1 | 0.0% | 119 | 3.4% | 464 | 13% | 1 | 0.0% | 282 | 8.1% | 585 | 17% |
| 2011 | 3,466 | 0 | 0.0% | 408 | 11.8% | 53 | 1.5% | 0 | 0.0% | 229 | 6.6% | 461 | 13% |
| Average | 3,593 | 135 | 3.7% | 208 | 5.6% | 151* | 4.6%* | 0 | 0.0% | 240 | 6.5% | 494 | 14% |

*3-year average, 2009-2011
