# Supplementary material for: Influenza-associated mortality in Thailand, 2006–2011
Source: Influenza Other Respir Viruses. 2015 Oct 13;9(6):298–304. doi: 10.1111/irv.12344 (PMC4605410; doi:10.1111/irv.12344)
Supplement: Supplementary file 4 — Table S3. Estimated average number of annual influenza-associated deaths with underlying P&I, respiratory, and circulatory causes by age group and location (without reapportion of ill-defined deaths). [file irv0009-0298-sd4.docx]

Supplement Table 3: Estimated average number of annual influenza-associated deaths with underlying P&I, respiratory, and circulatory causes by age group and location (without reapportion of ill-defined deaths)

| Underlying cause | Ages < 65 | | | | | | Ages > 65 | | | | | |
| --- | --- | --- | --- | --- | --- | --- | --- | --- | --- | --- | --- | --- |
|  | In hospital | | | Out of hospital | | | In hospital | | | Out of hospital | | |
|  | Death | 95% CI | | Death | 95% CI | | Death | 95% CI | | Death | 95% CI | |
| Respiratory disease | 427 | -927 | 1779 | 38 | -805 | 843 | 604 | -1565 | 2736 | 240 | -810 | 1171 |
| Pneumonia and Influenza | 264 | -702 | 1235 | 0 | -426 | 384 | 281 | -1117 | 1685 | 87 | -303 | 457 |
| Circulatory disease | 47 | -1797 | 1803 | 10 | -1226 | 1076 | 62 | -1949 | 1979 | 177 | -1367 | 1439 |
