# Supplementary material for: Influenza-associated mortality in Thailand, 2006–2011
Source: Influenza Other Respir Viruses. 2015 Oct 13;9(6):298–304. doi: 10.1111/irv.12344 (PMC4605410; doi:10.1111/irv.12344)
Supplement: Supplementary file 5 — Table S4. Estimated annual influenza-associated death rates in Thailand (without the reapportioned ill-defined deaths). [file irv0009-0298-sd5.docx]

Supplemental Table 4: Estimated annual influenza-associated death rates in Thailand (without the reapportioned ill-defined deaths)

| Cause of death | Influenza | | | | | |
| --- | --- | --- | --- | --- | --- | --- |
|  | Number of deaths | 95% CI | | Rate per 100,0000 | 95% CI | |
| Respiratory disease | | | | | | |
| <65 years | 465 | -1732 | 2622 | 0.8 | -3.0 | 4.5 |
| >65 years | 843 | -2375 | 3907 | 17 | -48 | 79 |
| All ages | 1308 | -4107 | 6529 | 2.1 | -6.5 | 10 |
| Pneumonia and influenza | | | | | | |
| <65 years | 264 | -1128 | 1619 | 0.5 | -1.9 | 2.8 |
| >65 years | 368 | -1420 | 2142 | 7.4 | -29 | 43 |
| All ages | 632 | -2549 | 3761 | 1.0 | -4.0 | 5.9 |
| Circulatory disease | | | | | | |
| <65 years | 56 | -3022 | 2879 | 0.1 | -5.2 | 4.9 |
| >65 years | 239 | -3316 | 3418 | 4.8 | -67 | 69 |
| All ages | 295 | -6338 | 6297 | 0.5 | -10 | 9.9 |
